# Nonenzymatic Reactions above Phospholipid Surfaces of Biological Membranes: Reactivity of Phospholipids and Their Oxidation Derivatives

**DOI:** 10.1155/2015/319505

**Published:** 2015-04-21

**Authors:** Christian Solís-Calero, Joaquín Ortega-Castro, Juan Frau, Francisco Muñoz

**Affiliations:** ^1^Institut d'Investigació en Ciències de la Salut (IUNICS), Departament de Química, Universitat de les Illes Balears, 07122 Palma de Mallorca, Spain; ^2^Instituto de Investigación Sanitaria de Palma, 07010 Palma, Spain

## Abstract

Phospholipids play multiple and essential roles in cells, as components of biological membranes. Although phospholipid bilayers provide the supporting matrix and surface for many enzymatic reactions, their inherent reactivity and possible catalytic role have not been highlighted. As other biomolecules, phospholipids are frequent targets of nonenzymatic modifications by reactive substances including oxidants and glycating agents which conduct to the formation of advanced lipoxidation end products (ALEs) and advanced glycation end products (AGEs). There are some theoretical studies about the mechanisms of reactions related to these processes on phosphatidylethanolamine surfaces, which hypothesize that cell membrane phospholipids surface environment could enhance some reactions through a catalyst effect. On the other hand, the phospholipid bilayers are susceptible to oxidative damage by oxidant agents as reactive oxygen species (ROS). Molecular dynamics simulations performed on phospholipid bilayers models, which include modified phospholipids by these reactions and subsequent reactions that conduct to formation of ALEs and AGEs, have revealed changes in the molecular interactions and biophysical properties of these bilayers as consequence of these reactions. Then, more studies are desirable which could correlate the biophysics of modified phospholipids with metabolism in processes such as aging and diseases such as diabetes, atherosclerosis, and Alzheimer's disease.

## 1. Introduction

Biological membranes play key roles in cell life, acting as permeability barriers and constituting privileged sites of communication between the inside and the outside of cells [1, 2]. The importance of membranes for life is reflected in the genomes, where around one-third of the coded information corresponds to membrane proteins [3]. The main structural element of biological membranes is a lipid bilayer, having other components, such as proteins, sterols, and peptides, either intercalated into or loosely attached to it [4]. This lipid bilayer has as primary role to define the permeability barrier of cells and organelles, having the hydrophobic domain of their lipid components oriented inward and shielded from water and their hydrophilic domain exposed and interacting with the aqueous environment [5]. This bilayer provides also the supporting matrix and surface for many enzymatic reactions, participating in signal transduction and providing precursors for signaling processes and macromolecular synthesis.

**Scheme 1:**
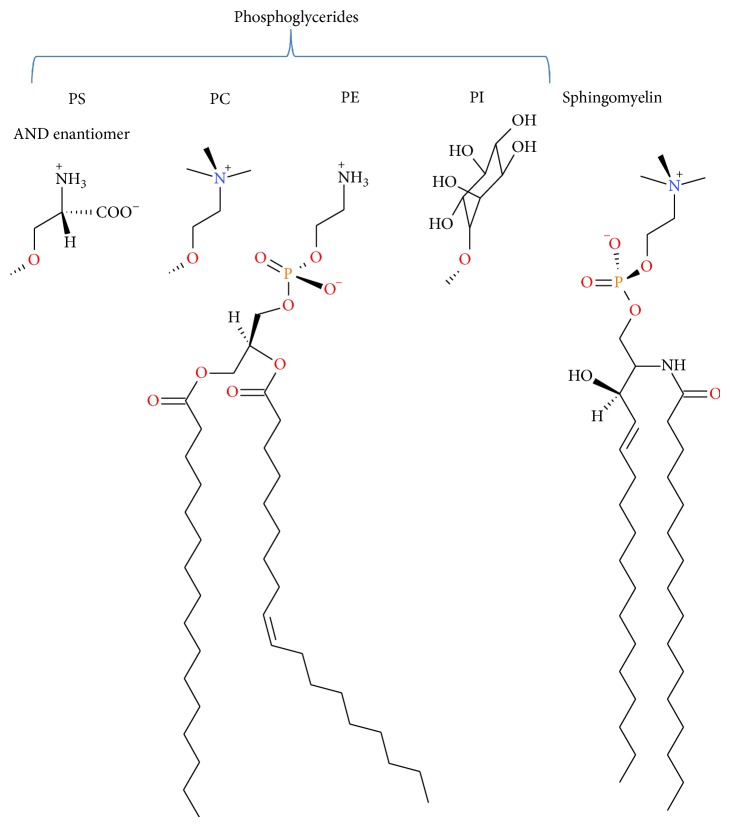
Structure of some phospholipids components of biological membranes (PS: phosphatidylserine, PC: phosphatidylcholine, PE: phosphatidylethanolamine, and PI: phosphatidylinositol).

Membrane lipid composition can vary dramatically across the three domains of life and even within single organisms [6]. In eukaryotic cells, phospholipids are the predominant membrane lipids (Scheme 1), phosphatidylcholine (PC) and phosphatidylethanolamine (PE) represent the 60%–85% of the phospholipid fraction, while the fraction of other phospholipids depends on the cell membrane and even on animal species [7, 8]. The distribution of phospholipids is asymmetric across the bilayer; nearly all anionic lipids in eukaryotic cells face the cytoplasm, whereas most lipids with large glycosylated headgroups are exposed to the extracellular environment. The chemical composition of the bilayer affects its mechanical properties and, conversely, the application of forces to the membrane can alter its chemical composition [9]. Phospholipids also serve as sources of arachidonic acid and other polyunsaturated fatty acids, which can be metabolized by oxygenase enzymes leading to the formation of various lipid mediators that regulate a variety of biological functions. Their nonenzymatic modification by lipid peroxidation and glycation could conduct to the formation of advanced lipoxidation end products (ALEs) and advanced glycation end products (AGEs). These modifications introduce changes in cell membrane physicochemical and biological properties and could be accumulated during aging and diabetes [7].

Evolution is very economical and it could use some cell components with an apparent main function in order to be useful for other purposes [10]. It is very known that proteins have had diversification of their functions in the course of evolution as a response to different environmental changes [11–13]; RNA molecules also have had different evolutionary developments [14] and although it is not yet found in nature, DNA has been shown to be able to catalyse transesterification reactions, in the same way of ribozymes and protein enzymes [15, 16]. As consequence of changes in these gene products, some ligands also have acquired diverse biological roles over the course of evolution [17, 18], and it is possible to hypothesize that other biological compounds could also diversify its functions. Phospholipids organized as a bilayer have as primary function to constitute a permeability barrier, being involved in a wide range of processes which explains the need for diversity in their structures. However, an inherent catalytic activity for phospholipids has not been suggested until few years ago [19–21], despite the charged and polar functional groups of the phospholipids heads which could act as proton donors and acceptors and the special dielectric medium constituted by the membrane/water interface.

**Scheme 2:**
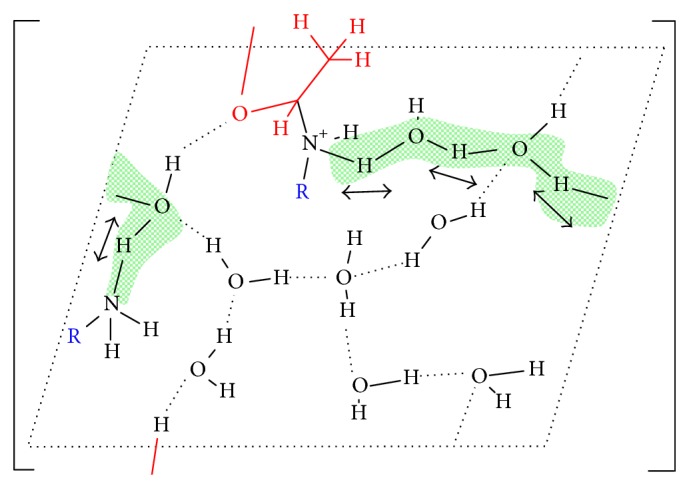
Transition state of reaction between PE (blue color) and acetaldehyde (red colour), showing the boundary translation invariance property of PE-surface periodic model. Arrows and green shadows indicate the direction and the protons involved in the transfer, dotted lines represent hydrogen bonds, and R corresponds to PE molecules which contain the amine groups.

**Figure 1:**
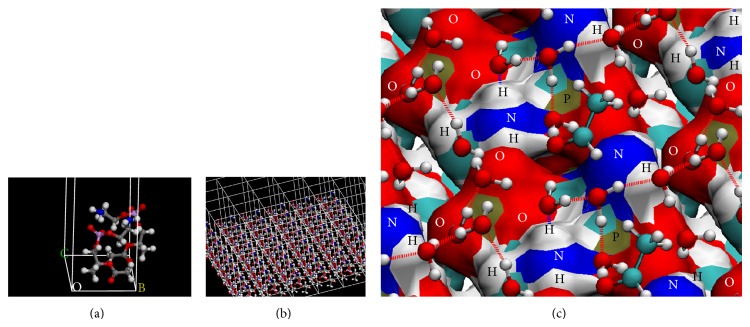
Model of phosphatidylethanolamine surface for study of Schiff base formation from PE and acetaldehyde. (a) Representation of cell unit of two molecules of truncated PE. (b) Extension of the cell by periodic boundary conditions. (c) A sight of PE surface, with acetaldehyde molecules and the water hydrogen-bond network. Atoms belonging to PE surface are labelled, and dotted lines represent hydrogen bonds.

Theoretical chemistry methods provide an atomistic-level description of the molecular systems, in a resolution that cannot be obtained experimentally, but their application in systems as biological membranes, which are enormously complex in terms of both structure and their dynamical properties, demands high computational power. In these cases, it is necessary to generate models of which nature and size depend on the studied property and the used level of calculus. By theoretical analysis, biological membranes properties have been mostly studied employing molecular mechanics-based methods and compared to first-principles computational studies [32–35]. There were only few studies using density functional theory (DFT) methods [36–38] but using cluster models for studying conformational properties generally. Our group at first obtained a mechanism for nonenzymatic reactions on a model of a phospholipid surface at DFT level of theory [19, 20, 22–24] (Table 1). The developed model of PE surface is a periodic model which has in its unit cell two PE molecules according to the studied reaction and a sufficient number of water molecules for forming hydrogen-bond networks (Figure 1). The procedure for obtaining the mechanisms of reactions included calculating transition states and describing energetic and geometric changes along the reaction coordinates, features difficult to describe based on experimental results only. Periodic boundary conditions were fundamental for these calculations, making it possible to model a phospholipids surface, studying its reactivity at the DFT level, and allowing the equivalent transfer of protons between unit cell images due to their boundary translation invariance property (Scheme 2). This methodology has been applied extensively in theoretical modelling of solid-state materials [39, 40] and little to biological molecules [41].

Other theoretical methods used for studying lipid bilayer systems are classical molecular dynamics simulations, which are able to provide more complete description of the dynamics and energetics of membranes with high spatial-temporal resolution [4, 42, 43]. In these methods, the positions of individual atoms in the system are followed by numerically solving classical equations of motion. Potential energy of the interactions is described in the form of force field, based on both empirical and quantum chemical data [43, 44]. Since molecular dynamics simulations are based on models, the results of such simulations are required to be validated by experimental data; a good agreement between them makes it reasonable to trust the basic models and use the simulations to explain experimental results and extend the analysis to phenomena that cannot be studied by experiments [43, 45]. Despite that, these methods have some limitations, originated by uncertainties in the chosen force field, and the short accessible simulation times, being not possible, match the kinetics of the studied process by the time span simulated.

**Figure 2:**
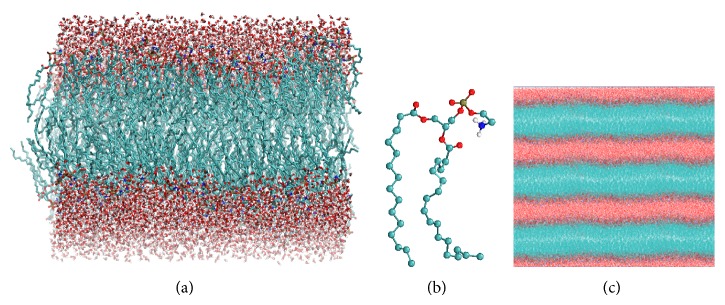
A simple model of 1-palmitoyl-2-oleoyl-phosphatidylethanolamine (POPE) bilayer. (a) Large POPE bilayer with 340 phospholipids and water molecules on its surfaces. (b) Backbone of POPE molecule. (c) Periodic boundary conditions in three dimensions of POPE bilayer model.

An additional limitation in membrane models is the size of the models; it is limited to several nanometres. Thus, no phenomena happening at longer length scales can be studied, which includes, for instance, the creation of large water defects and pores [44]. Initial models of biological membranes were simple hydrated PC bilayers systems; the complexity of these models was gradually increased by successive addition to the bilayer of other natural membrane components like other phospholipids, cholesterol, peptides, and proteins (Figure 2) [43]. The necessity for probing larger simulations time and length scales of systems has been tried to be solved using coarse grained models of molecular systems as phospholipid bilayers [26, 46, 47]. These models represent the molecular systems by a reduced number of degrees of freedom, clustering groups of atoms into coarse grained sites, or beads, which lack atomic details but keep the critical features of the molecules which determine the physics of the length scale under study [46, 48]. In relation to studies of reactivity, all these methods at classical molecular mechanics level of calculus cannot be used for studying this kind of processes. However, in the case of membrane models, the results of simulations could lead to analyzing changes in the molecular interactions and biophysical properties of bilayers systems, by the introduction of some products of reactions into them that occurs on phospholipids (Table 2) [25–31].

This review is organized as follows. A description of the phospholipids reactivity as part of biological membranes is summarized first. The activity of reactive oxygen species (ROS) on phospholipid membranes, how ROS access them, and the oxidation reactions which yield reactive carbonyl species (RCS) are reviewed next. A brief description of generation of advanced lipoxidation end products (ALEs) and advanced glycation end products (AGEs) and their consequences on biophysical properties of membranes are also reviewed. Some general ideas and hypotheses about the biophysical impact of the modification of phospholipid membranes and the reactivity of different functional groups of phospholipids derived from theoretical studies are presented in all the items of this review.

## 2. Phospholipid Reactivity

The first comprehensive model of the biological membranes, the “fluid mosaic model,” assumes the lipid bilayer to be a passive structure fulfilling two basic functions: supporting proteins and forming a barrier for dissolved molecules in the aqueous phase [49]. However, experimental data obtained in studies on biological and model membrane systems made lipid bilayer be also recognized as a potent enhancer and regulator of surface associated reactions [50–52]. In order to study lipid bilayer's structural complexity, Tieleman et al. proposed to divide the membrane into four different regions [43]. The second region is an interface which begins where water density is equal to that of headgroups and reaches the level of carbonyl groups. The interface also includes bounded ions and organized water molecules not easily to be described with macroscopic theories. This apparent simple interface of one componential lipid bilayer is actually a region with complex structure, sensible to changes in its physicochemical properties as a result of changes in its composition. For example, when water concentration decreases, dielectric permittivity changes from nearly 80 to about 2 (over a distance of about 1 nm [53]), and dipole potential affects electrostatic potential at the immediate vicinity of the membrane or within the interface [50].

The membrane surfaces are so complex, having developed some electrostatic models for describing their properties. One of them is Gouy-Chapman electrostatic theory which relates the charge density and electrical potential at the surface of a membrane to the concentrations of ions in the external solution, treating the interface as a charged structureless plain surrounded by uniform environments [50, 54]. Actually, the membrane charges are not smeared-out uniformly over the surface and the transition between the bulk solvent and the membrane interior is not discontinuous. The membrane charges are always discrete and distributed throughout a mesh-like, water-containing region. Their arrangement depends on the type of lipid present in the membrane. The variety of lipids and their biological functions suggest that charge distribution determines the extent and type of interaction with surface associated molecules [50]. The solvent properties and ion concentration in this region may differ largely from the bulk, and they also could be modified taking into account possible appendages in the surface, which range from glycolipid and protein molecules, with dimensions on the order of a few nanometers, to cilia and microvilli, on the order of micrometers [54].

The amount of water and its organization and mobility change with its distance from the aqueous phase to the surfaces [43, 55–57]. Particularly, the organization of water around charged surfaces greatly affects effective electrostatic potential. Zwitterionic headgroups of phospholipids create dipole and higher multipole fields which, in principle, could be relevant for the membrane hydration [54, 58]. Theoretical studies have shown that water molecules close to a polar surface are oriented by an electrostatic field associated with lipid residual charges [59, 60]. Moreover, water-membrane association has been inferred to be governed by charge-transfer rather than by charge-charge interactions, being one source of such effects, the strong local interactions such as atomic or dipolar-fields near the individual charged or polar groups at the membrane surface [54]. These interactions are also involved in the hydrogen bonds or other charge-transfer processes between the water molecules and the membrane constituents and/or between themselves.

The particular chemical environment of this interface makes phospholipids surface interact with not only small molecules [61–64] but also macromolecules [65–67], via electrostatic and hydrophobic interactions and/or hydrogen bonding. The cell membrane surface could also enhance the ability of protons to diffuse promptly along the membrane through hydrogen-bonded networks of water molecules and charged or polar groups of phospholipids at the surface [68]. The membrane-buried layers of these networks can eventually serve as a storage/buffer for protons (proton sponges) [69, 70]. Phospholipid components of cell membrane also exhibit particular reactivity in this interface, which has been evidenced experimentally [21, 71–76] and theoretically [19, 20, 22, 23, 77]. Several of these experimental and theoretical studies suggest the catalytic potential of phospholipids surface for participating in other reactions that occurs on cell membrane surface. Beyond surface charge, the phospholipid headgroups may also affect other physicochemical properties of phospholipid bilayers, such as packing of the lipid chains, the thermotropic phase behavior, and the isothermal lateral phase separation of the bilayer into discrete domains of different compositions [78].

**Figure 3:**
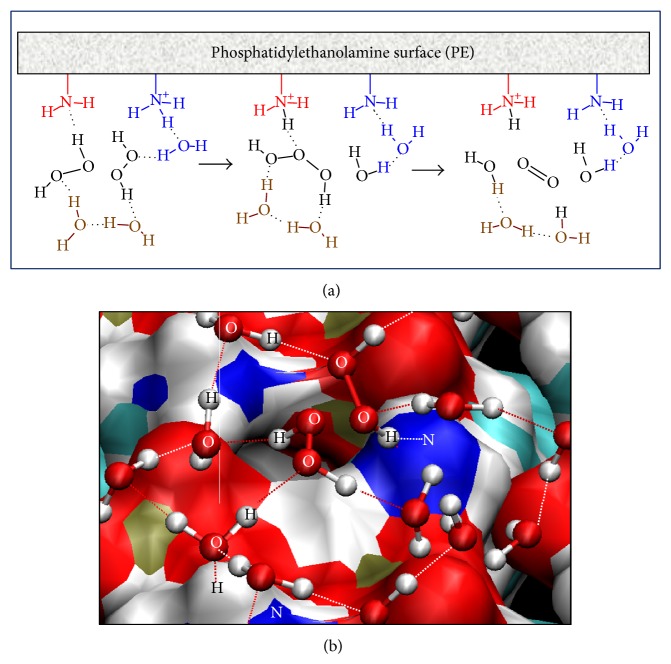
Pathway for hydrogen peroxide decomposition on PE surface. (a) Scheme of two steps of reaction, formation of an intermediate hydrogen trioxide from two H_2_O_2_ molecules and its hydrolysis in O_2_ and H_2_O. (b) Starting structure of modelled reaction, including two molecules of hydrogen peroxide, a PE surface, and solvent molecules, forming a hydrogen-bond network. Atoms which participate in the reaction are labelled; dotted lines represent hydrogen bonds.

Phosphatidylethanolamine (PE) is recognized as one of the main components of biological membranes, being one of the most abundant lipids in eukaryotic cell membranes unevenly distributed between the inner and the outer leaflets of the bilayer [79]. Its presence under possible primitive earth conditions was also hypothesized [80] and also performs many biological roles beyond its structural role, having, for example, a contribution to apoptosis and cell signaling [81–83], as donor of the ethanolamine moiety that covalently modifies several proteins [84, 85]. Our theoretical studies about several reactions on PE surface (Table 1) attributed an additional role to PE, a catalytic role through its amine and phosphate groups in the context of a model which simulate the interface water/PE-monolayer [19, 20, 22, 23, 77]. This PE-surface model is a three-dimensionally periodic slab model. In all the studied reactions, the supercells contained two molecules of truncated PE, additional reactive molecules, and sufficient number of water molecules as explicit solvent in a hydrogen-bond network along the polar heads of phospholipids. The periodic boundary conditions made it possible to obtain a surface model of a layer of phospholipids, useful for studying theoretically the reactions on an environment different to aqueous solvent (Figure 1). The catalytic effect of PE surface was interpreted in three ways: (i) accumulation of water molecules on the surface, increasing their organization, reducing their mobility, and polarizing their bonds as a result of an interaction with the PE charged groups; (ii) direct acid catalysis through amine groups of PE molecules adjacent to reactive PE molecule acting as donors or acceptors in the proton transfers, and water molecules in the surface surroundings acting as bridges in the proton exchange between donor and acceptor protons in the reaction (Scheme 2 and Figure 3); (iii) passive catalytic effect through a charge stabilization of different intermediate structures of reaction, due to direct electrostatic interactions with the charged groups generated in the different steps of the reaction. In this particular chemical environment, the role of water molecules in the direct proximity of PE surface was not only as solvent, if not, it is also catalytic, acting as bridge between proton donor and acceptor groups and stabilizing electrostatically transition states and intermediates structures through hydrogen bonds with charged and polar groups of them. These results were in agreement with experimental data about these reactions [21, 86] and concluding in the catalytic potential of phospholipids surfaces for participating in other reactions that occur on the cell membrane surface.

It is known that considering surface as a chemical environment for biochemical reactions brings changes in their characteristics [50]. Membrane-associated reactions are controlled by the transmembrane and lateral distribution of reactive molecules, which depends ultimately on the state of the lipid bilayer and the efficiency of its transport mechanisms. On the other hand, biochemical reactions occurring in aqueous solutions are controlled almost exclusively by diffusion [50]. In the studied reactions on PE surface, the catalytic effect of the surface was evidenced through the reduction of free energy barriers in the reaction pathways in comparison with the same reactions studied in pure water models, such as Schiff base formation, Amadori rearrangement, and hydrogen peroxide decomposition [19, 20, 22, 77].

Although phosphatidylserine (PS) represents a quantitatively minor phospholipid in mammalian cells, the presence of PS is also required for many fundamental cellular processes. Its distribution is asymmetrical in the plasma membrane, constituting ~25% of the phospholipid in the inner leaflet, what carries an appreciable negative charge above the cytosolic surface of the plasma membrane, in comparison with other intracellular membranes, creating a chemical environment where special biochemical process occurs [87, 88]. It is known that changes in the PS distribution have strong consequences when PS molecules naturally flip to the outer surface; they act as a signal for macrophages to phagocyte the cells [89, 90]. By itself, PS keeps several described reactivity properties described for PE, participating in analogous reactions [91, 92]. However, PS participates in many intracellular processes depending on its anionic nature. For example, some key signaling proteins, such as the tyrosine kinase and Src as well as the Ras and Rho family of GTPases, contain positively charged motifs that bind to PS [93–96]. Electrostatic attraction between anionic groups of PE and positively charged regions on proteins has been suggested to contribute as a driving force for the plasma membrane localization of these peripheral proteins [87].

Phosphatidylcholine (PC) is the major phospholipid in mammalian membrane and is synthesized in the liver via the choline pathway by methylation of PE via phosphatidylethanolamine N-methyltransferase [97]. In addition to its function as a membrane constituent, PC has a role in signalling via the generation of diacylglycerols by phospholipase C, being also precursor for phosphatidic acid, lysophosphatidylcholine, and platelet-activating factor, each with important signalling functions, and other structural lipids such as sphingomyelin and PS. PC in its form of dipalmitoylphosphatidylcholine (DPPC) has been considered the benchmark lipid in the study of model bilayers, experimentally and theoretically [43]. The hydration of the headgroups is probably the most drastic difference between PC and PE as component of cell membranes; around the PC headgroup, the water molecules orient themselves in a clathrate structure, characteristic of solvation shells of hydrophobic solutes. On the other hand, the PE headgroup solvation involves hydrogen bonding with water molecules and other PE headgroups [98, 99]. This difference is attributed to the larger hydrogen-bonding possibilities of the PE headgroups with easily available polar hydrogens, in comparison with the PC headgroup with bulky methyl groups instead [43]. The same structural features make PC not exhibit the same reactivity of PE. However, a catalytic effect of PC on hydrogen peroxide decomposition has been suggested based on experimental results using PC vesicles [21].

Knowing the catalytic potential of phospholipids surface could be also important in the emerging field of synthetic biology [100], for constructing novel biological systems, which function in a robust and predictable manner in novel biological contexts, useful as biotechnological tools, biomedical devices, or biofunctional materials [101]. It has been shown that some PE-based liposomes of various compositions are stable and pH-sensitive [102–104]. An acidification of the environment leads to membrane destabilization, fusion, and release of entrapped aqueous vesicle contents, being for that useful for the delivery of foreign substances as drugs into living cells. They have been tested as an efficient drug delivery system in the treatment of malignant tumours [105]. PE conjugated with hydrophilic polymers as polyethylene glycol forms polymeric micelles which have also been useful as drug delivery systems [106–108].

## 3. Reactive Oxygen Species and Phospholipid Membranes

Reactive oxygen species (ROS) are generated from the metabolism of molecular oxygen and participate in redox signaling pathways that are essential for the physiological control of cell function [109, 110]. However, they are also involved in the process of aging and several chronic diseases such as atherosclerosis, diabetes, Alzheimer's disease, asthma, rheumatoid arthritis, and many forms of cancer, being also generated by numerous extracellular agents such as pollutants, tobacco smoke, iron salts, and radiation [110, 111]. ROS include free radicals such as superoxide radical (O_2_^−^), nitric oxide radical (NO^•^), hydroxyl radical (OH^•^), and hydroperoxyl radical (HOO^•^) as well as nonradical molecules like hydrogen peroxide (H_2_O_2_), ozone (O_3_), trioxidane (HOOOH), and nitric oxide (NO) [109, 110]. Radical species such as OH^•^ and, to a lesser degree, O_2_^−^ tend to be regarded as more reactive and poorly selective, whereas many nonradical species such as H_2_O_2_ show more selectivity in their reactions with biomolecules being able to migrate in aqueous medium freely [112].

The vulnerability of membrane phospholipids to be damaged by ROS is related to the physicochemical properties of the membrane bilayer and the chemical reactivity of the fatty acyl chains composing the membrane [113]. The first property is related to the fact that oxygen and free radicals exhibit more solubility in the fluid lipid bilayer than in the aqueous solution. The values of solubility of O_2_ are approximately 20–30 more than aqueous media [114], making it readily available to fuel these reactions in a lipid environment, where it also increases its reactivity in comparison with the surrounding aqueous medium [115]. On the other hand, classical molecular dynamics simulations have shown that hydroxyl and hydroperoxyl radicals were able to penetrate deep into the lipid headgroups region, having access to potential peroxidation sites along the lipid hydrocarbon chains, without having to overcome the permeation free energy barrier [25]. In this point, it is necessary to take into account the case of short-lived radicals such as OH^•^, where its steady-state is a result of competing processes of radical generation and scavenging rather than an equilibrium distribution over larger distances. The hydroxyl radical (OH^•^) diffuses in aqueous solution via H-transfer reactions with solvent molecules which has been shown by Car-Parrinello molecular dynamics [116]. The solubility of other ROS such as superoxide (O_2_^−^) radical and hydrogen peroxide (H_2_O_2_) is less in the fluid lipid bilayer, remaining at the aqueous phase.

**Scheme 3:**
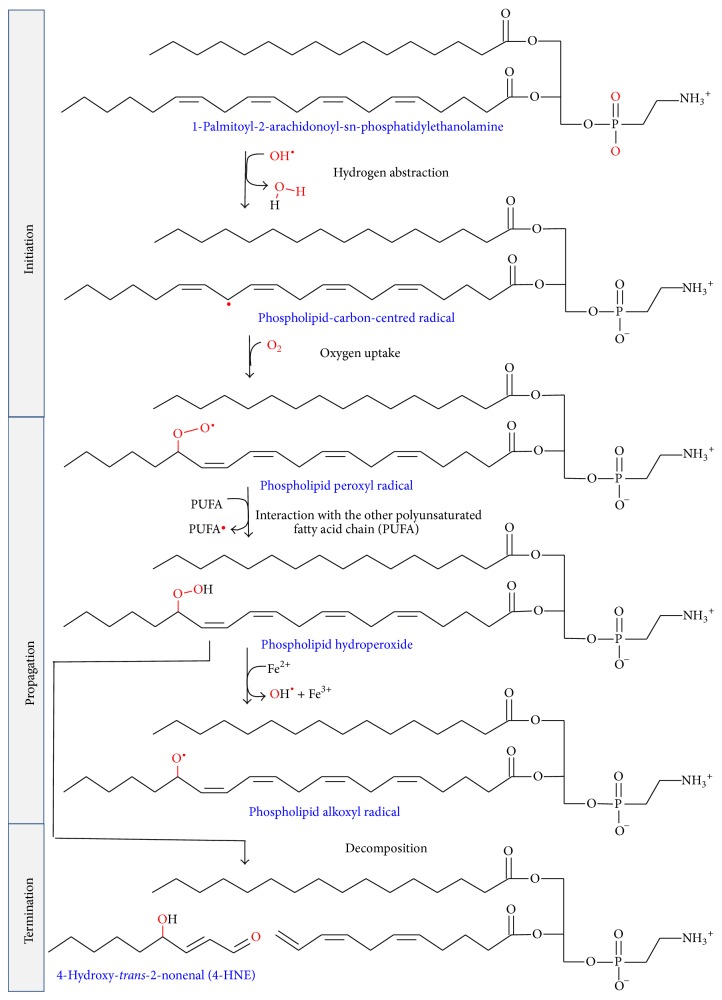
Scheme of the steps in lipid peroxidation of 1-palmitoyl-2-arachidonoyl-sn-phosphatidylethanolamine.

**Scheme 4:**
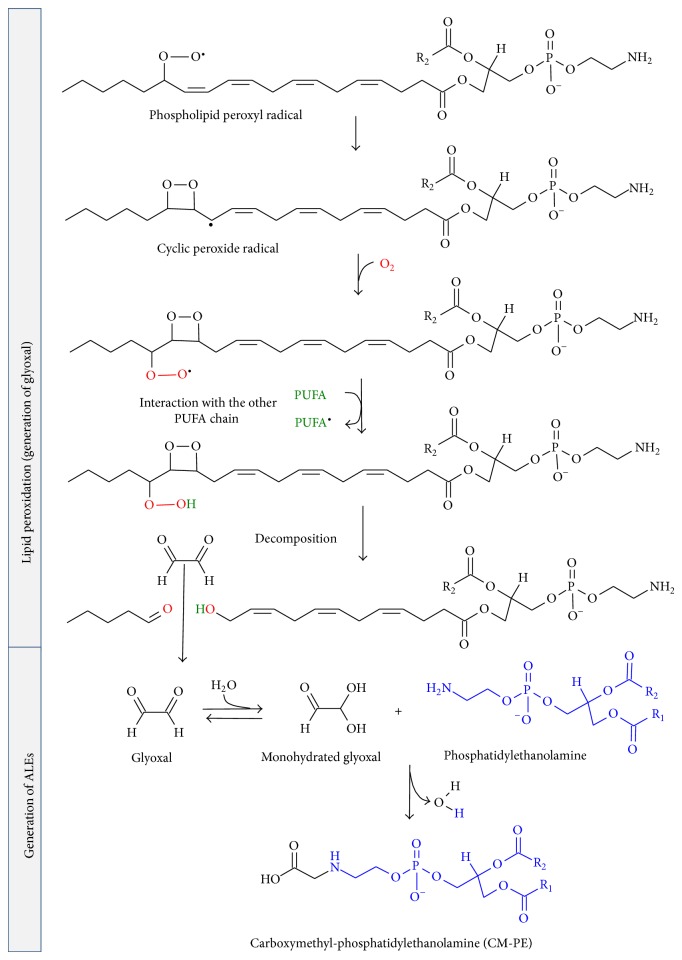
Generation of ALEs from phospholipid peroxidation, showing possible routes to formation of glyoxal and subsequent CM-PE. R_1_, R_2_ correspond to fatty acyl chains of phospholipids.

**Scheme 5:**
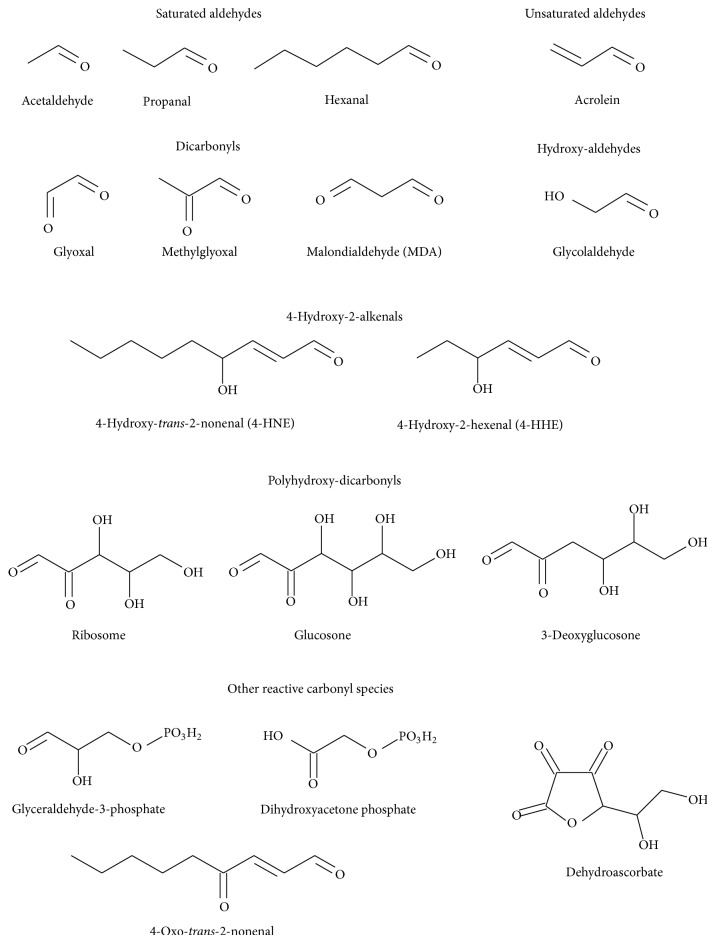
Chemical formulae of most common biological reactive carbonyl species.

Once ROS are inside biological membranes, in particular, free radicals ROS are able to induce peroxidation of membrane lipids, above all in membranes composed of residues of phospholipids with polyunsaturated fatty acids (PUFAs) [117]. PUFAs are generated by action of desaturases on saturated fatty acids, being essential components of cellular membranes in higher eukaryotes, but, at the same time, they are extremely sensitive to oxidation [113]. Then, in comparison with other peroxidation processes, PUFA peroxidation has a self-propagating nature, because abstraction from a methylene group of a hydrogen atom has as a result an unpaired electron on the carbon, (–^•^CH–). C–H bonds on the carbon atom nearby the PUFA double bond are weakened by its close presence, facilitating the hydrogen subtraction [117, 118]. In general, lipid peroxidation reaction has three steps: initiation, propagation, and termination (Scheme 3). The initiation step between ^•^OH radical and polyunsaturated fatty acyl chains generates a lipid radical (L^•^), which in turn reacts with molecular oxygen to form a lipid peroxyl radical (LOO^•^). Propagation step starts with a hydrogen abstraction by LOO^•^ from an adjacent fatty acyl chain, producing a relatively stable lipid hydroperoxide (LOOH) and a second lipid radical [119]. Then, reduced metals, such as Fe^2+^, can cleave reductively LOOH, producing lipid alkoxyl radical (LO^•^). Both peroxyl (LOO^•^) and alkoxyl (LO^•^) radicals are able to propagate lipid peroxidation by abstracting additional hydrogen atoms [120]. Termination step includes the fragmentation of LOOH, in the presence or absence of reduced metals or ascorbate, into a large variety of reactive intermediates called reactive carbonyl species (RCS) with three to nine carbons in length (Schemes 3 and 4). RCS (Scheme 5) include very reactive *α*,*β*-unsaturated aldehydes such as 4-hydroxy-trans-2-nonenal (4-HNE), 4-hydroxyhexenal (4-HHE), and acrolein, dicarbonyls such as malondialdehyde (MDA) and glyoxal, and keto-aldehydes such as 4-oxo-trans-2-nonenal (ONE) and isoketals [113, 119, 121]. Oxidation of n-6 PUFAs leads to the formation of 4-HNE, whereas oxidation of n-3 PUFAs generates 4-HHE [122]. Phospholipids can also be attacked by reactive nitrogen species (RNS) and chlorine species, further expanding the range of products to nitrated and chlorinated phospholipids [112].

Resulting oxidized phospholipids are able to modify the function and physical properties of the membranes, including their fluidity, permeability to different solutes, bilayer thickness, and the packing of lipids and proteins in the membranes [78]. Experimental and theoretical studies on model membranes with introduced oxidized lipids demonstrated that cell membrane damage by oxidative stress causes alteration of water penetration in the bilayer, due to an increase in polarity and hydrogen-bonding propensity in the central region of the bilayer [28]. The oxidized lipid tails have the tendency to bend toward the water interface, because they are more polar and can be shorter in length, due to the presence of aldehyde or hydroperoxide groups. This tendency is responsible of the changes in the structural properties of the lipid bilayers, increasing their water permeability, which lead to an increase in the average area per lipid and, correspondingly, to a decrease of the bilayer thickness [28, 29, 31]. These changes enhance the susceptibility of the membrane to electropermeabilization, a procedure which has as application the introduction of genetic material and pharmaceutical agents into living cells [30]. On the other hand, the accumulation of lipoperoxidation products as malondialdehyde is able to disturb the aminophospholipids organization in the human erythrocyte membrane bilayer, compromising its integrity [123]. The membranes composition could influence the degree of damage caused by phospholipid oxidation. A coarse grained molecular dynamics study attributes to cholesterol a protecting role on bilayers from disrupting agents as oxidized phospholipids [26]; this role is related to the cholesterol rearrangement in the oxidized membrane exhibiting a preferable interaction with carbonyls of the oxidized chains [29].

Generally, phospholipid headgroups are not affected by peroxidation reactions. However, these headgroups could influence the rates of peroxidation which has been shown in studies using phospholipid liposomes [78]. There is experimental and theoretical evidence that H_2_O_2_ decomposition is accelerated above phospholipids membranes surface [20, 21], having in this way an indirect protective effect against the possible damage caused by this nonradical ROS. Our group developed a mechanism of this reaction by two steps (Figure 3(a)). In the first step, an intermediate hydrogen trioxide from two H_2_O_2_ molecules is formed. In the second step, this intermediate is cleaved in O_2_ and H_2_O [20] (Figure 3(a)). The results have also shown the first step as the limiting step of the reaction, having a free energy barrier of 8.76 kcal mol^−1^ in comparison with the same reaction in a pure water model where this barrier had a value of 25.56 kcal mol^−1^. This catalytic effect of PE surface was done directly by amine groups of PE and water molecules in the proximity of PE surface being part of the hydrogen-bond network. The two PE molecules of the system acted like proton donor and acceptor, assisting directly the reaction in the deprotonation of the first H_2_O_2_ molecule (Figure 3). The role of components of PE surface was not only limited to participate in proton transfer, if not also favouring accumulation of the main reactive H_2_O_2_ on the proximity of the surface. The phosphate anion of phospholipids forms hydrogen bonds with water molecules in the network connecting donor and acceptor protons (Figure 3(b)); it could facilitate accumulation of H_2_O on the membrane surface and in consequence of H_2_O_2_ in its proximity, raising local concentrations of the last as a result. The H_2_O_2_ decomposition on phospholipids surfaces could contribute to other biochemical mechanisms such as natural antioxidant molecules as ascorbate [124, 125] and antioxidant enzymes [126, 127] for regulating the concentration of H_2_O_2_, being able to cause biological damage. It is also essential for functions related to signal transduction, cell growth enhancement, cell proliferation, cell differentiation, and apoptosis at moderate concentrations [128–132].

## 4. Advanced Lipoxidation End Products (ALEs)

The reactive carbonyl species (RCS), mainly short-chain aldehydes (Scheme 5) generated during lipid peroxidation, have longer half-life values in comparison with ROS or RNS, being able to migrate through hydrophobic and hydrophilic media due to their polar and noncharged structure. RCS are electrophilic and therefore highly reactive toward nucleophilic groups in biomolecules such as proteins, DNA, and aminophospholipids. Unsaturated RCS are usually an order of magnitude more reactive than their saturated counterparts [133, 134]. These reactions result in the irreversible modification of biomolecules through the formation of a variety of adducts and cross-links collectively named advanced lipoxidation end products (ALEs).

Generation of ALEs involves the loss of function and structural integrity of modified biomolecules and may be the cause of subsequent cellular dysfunctions and tissue damage. In the proteins, their strong nucleophilic sites such as thiol, imidazole, and hydroxyl groups are the most attractive targets for electrophilic attacks of RCS. As an example, 4-HNE can react with histidine (His), cysteine (Cys), or lysine (Lys) residues of proteins, leading to the formation of stable Michael adducts with a hemiacetal structure [135]. The factors that can affect selectivity of oxidative damage to proteins could include their values of the half-life, their rate of proteolysis, their molecular conformations, presence of a metal-binding sites, and relative abundance of amino acid residues susceptible to oxidative reactions [113]. RCS can also react in the exocyclic amino groups of nucleotides to form various alkylated products, deoxyguanosine being the most commonly modified DNA base, because of its high nucleophilicity [136].

Aminophospholipids such as PE and PS can also react through their primary amino groups with RCS carbonyl compounds, obtaining analogous products from reactions between RCS and proteins. PE was found to be a good target for a reaction with 4-HNE, having as main product a Michael adduct which could be partly cyclized as a pyrrole derivative via a loss of water. On the other hand, PS reacted with 4-HNE poorly producing only a small amount of Michael adduct [137–139]. Isoketals have been also shown to react with PE, forming pyrrole adducts and Schiff base adducts [140]. Excessive modification of plasma membrane aminophospholipids with the formation of ALEs modifies the composition of biological membranes, altering the asymmetrical phospholipid membrane distribution, resulting in changes in the membranes physical properties as fluidity which are translated in changes in membrane structure and function [136]. As examples, this membrane damage could alter the processes of biosynthesis and turnover of membrane phospholipids and the activity of membrane-bound proteins that require aminophospholipids for their function [136].

**Scheme 6:**
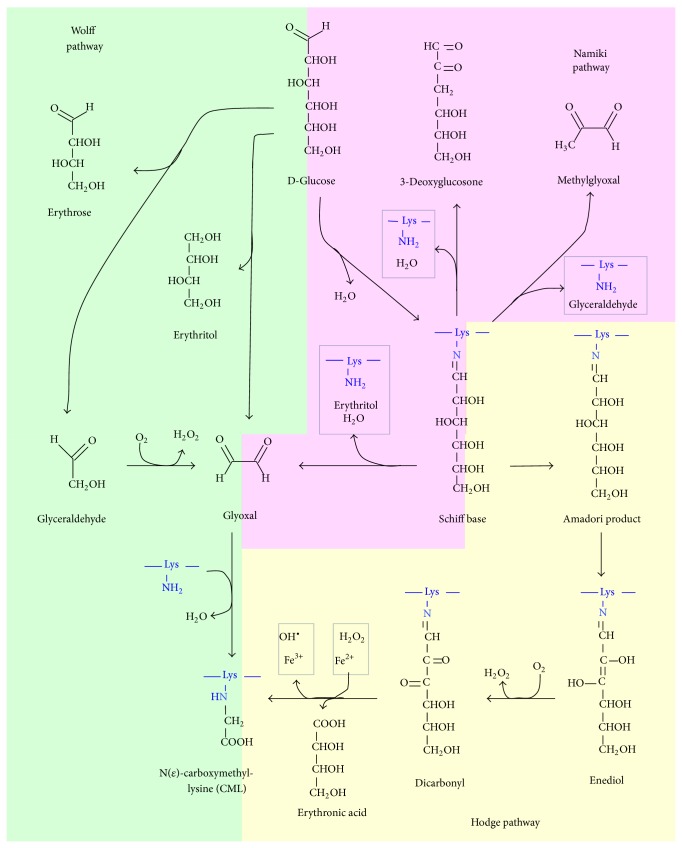
Pathways leading to AGEs formation in proteins. They include Wolff, Namiki, and Hodge pathways, being also possible routes to formation of phospholipids AGEs.

Some RCS such as glyoxal and methylglyoxal are generated also by sugar oxidative degradation pathways (Scheme 6), which include autoxidation reactions and Maillard reaction; thus, the corresponding reaction products with proteins or phospholipids can be named both as ALEs and AGEs or as EAGLEs (either advanced glycation or lipoxidation end products) [141]. Glyoxal could also be generated by myeloperoxidase-mediated degradation of serine at sites of inflammation and hydrolysis of ascorbate [142, 143]. The amino group of aminophospholipids can also react with these carbonyl compounds and initiate some of the reactions occurring in proteins and DNA, leading to the formation of adducts such as carboxyethyl-PE and carboxymethyl-PE (CM-PE) (Scheme 4) [144, 145]. The CM-PE generation in human erythrocytes and blood plasma has been demonstrated experimentally [145–147]. It is possible to hypothesize that the generation of CM-PE could proceed by similar mechanisms compared to CML (Scheme 6) [145, 148, 149]. Our group has obtained, using DFT calculations at DFT level of theory, a mechanism of formation of CM-PE from reaction between glyoxal and PE [23]. It has been hypothesized that carboxymethyl-PE could trigger pathological processes [150, 151], being suggested due its relationship with Maillard reaction, as a potentially sensitive marker for reflecting hyperglycemic conditions in the early stage of diabetes [145, 152].

The rates of oxidation and aldehyde adduct formation are low under physiological conditions [122]. However, they are increased during aging by a progressive decline in mitochondrial function which results in the accumulation of ROS [153, 154]. The rates of aldehyde adduct formation and glycation are also different between the different biomolecules with free amine groups, these rates being influenced by the half-life of them. In the case of proteins, there is a rapid turnover of short half-life of cellular proteins, there are proteolytic systems which ensure the removal of modified proteins, and proteasomal degradation is activated by oxidative stress, but their activities decline during the aging process, leading to the accumulation of damaged proteins and the formation of protein aggregates [155, 156]. Moreover, there are a number of intracellular proteins such as elastin, eye lens crystallins, and several collagens, which evade the turnover process and instead are maintained on the order of years, being prone to accumulation of damage by oxidants and glycating agents [157, 158].

The half-life of phospholipids is consequence of a constitutive recycling of membrane phospholipids by a complex biochemical system, and it is more responsible to protect membranes from lipid oxidative damage than selective in situ repair [159]. The phospholipids half-life values depend on the cell type and cellular location; in the case of rat liver, an experimental research attributes values from 22 to 36 hours for the half-life of subcellular phospholipids [160]. According to experimental results, in rat brains the half-life of PE is 19.5 days taking into account the whole brain and 11.5 and 14.5 days considering only neurons and glial cells, respectively [161]. The diversity of turnovers for phospholipids increases if we take into account only molecular parts of them such as their fatty acyl chains, not sensible to attack of RCS or reducing sugars but sensible to ROS. In the n-3 PUFA adequate rat brains, [4,5-3H]docosahexaenoic acid (DHA) has half-lives equal to 33 days in total brain phospholipid, and this time is prolonged by 15 weeks of nutritional deprivation of n-3 PUFA [162]. On the other hand, half-life for arachidonic acid from brain phospholipids is not altered by deprived diet, suggesting a different mechanism in comparison with DHA [163].

## 5. Advanced Glycation End Products (AGEs)

**Scheme 7:**
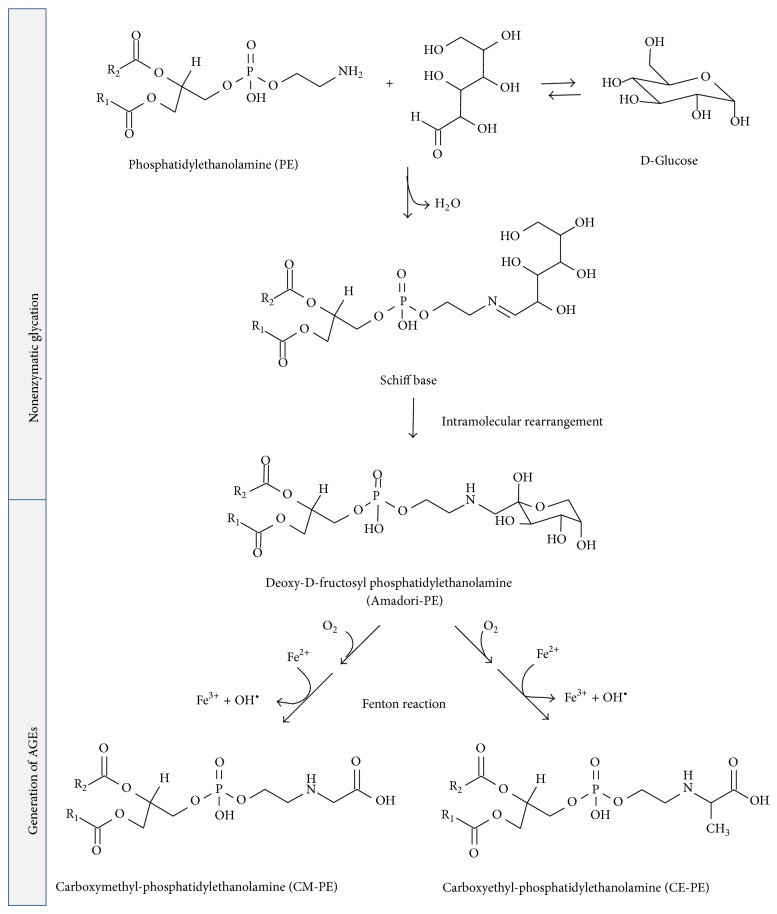
Generation of AGEs from glycation of PE, showing possible routes to formation of CM-PE and CE-PE. R_1_, R_2_ correspond to fatty acyl chains of phospholipids.

Advanced glycation end products (AGEs) are a complex and heterogeneous group of compounds, resulting from various pathways. One of them is a process known as nonenzymatic glycation, which includes a series of slow reactions between the carbonyl groups of reducing sugars such as glucose, fructose, or pentose and the amino groups of several kinds of biomolecules to yield an unstable Schiff's base followed by generation of stable ketoamine known as Amadori product (Scheme 7). The formation of Schiff's bases depends on the amount of reducing sugars and free amino groups of the biomolecules as well as an alkaline pH value. In contrast, Amadori rearrangements proceed at an acidic pH value and have a slower reaction rate [164]. Lastly, under certain conditions as high concentrations of glucose, oxidative and nonoxidative processes result in rearrangements and fragmentations of Amadori products into the irreversible AGEs (Hodge pathway) [165, 166]. Formation of AGEs could also proceed from cleavage of Schiff's base intermediates (Namiki pathway) [167] as well as the formation of carbonyl compounds after autoxidation of monosaccharides, such as glucose, ribose, fructose, and glyceraldehyde (Wolff pathway) [164, 168] (Scheme 6).

Our group has experimental and theoretical background about Schiff bases formation and Amadori rearrangement mechanisms in different molecular systems [19, 22, 73, 74, 77, 169–172]. A mechanism for the formation of Schiff base from acetaldehyde and PE on a model of phospholipids surface based on DFT calculations takes place in two steps, a formation of a carbinolamine intermediate, followed by its dehydration to Schiff base, with the dehydration being the rate-determining step of the process (Table 1), consistent with available experimental evidence for similar reactions [77]. The mechanism of the formation of the Amadori product from Schiff bases resulting from reactions between D-erythrose and PE has been studied [22]. The mechanism started with the formation of a 1,2-enaminol intermediate, followed by its ketonization to Amadori product (Scheme 7). The most important outcome of these studies was highlighting the catalytic role of PE surfaces on the reactions which proceed above them. It has been described in the item about aminophospholipids surface reactivity of this review. Moreover, the obtained mechanisms could be extrapolated to the reaction having glucose or other reducing sugars instead of D-erythrose, contributing towards better understanding a part of nonenzymatic glycation on phospholipid surfaces. In both mechanisms, all the reactions were governed to a great extent by the network of hydrogen bonds formed between water molecules on PE surface and the polar and charged groups of PE and acetaldehyde or D-erythrose (similar to that obtained in Figure 1). Direct interactions between sugars and the lipid headgroups through the formation of hydrogen bonds have been demonstrated experimentally and through molecular dynamics simulations [34, 173–176].

Nonenzymatic glycation of proteins known as Maillard reaction is increased in diabetes mellitus patients and leads to several complications such as blindness, heart disease, nerve damage, and kidney failure [177]. One of the major AGEs of proteins widely found* in vivo* is the N*ε*-(carboxymethyl)lysine (CML), which can be formed by three concurrent mechanisms* in vivo* [178]. The relationship with some diseases has been documented extensively [106, 179, 180]. In the classical Hodge pathway, Amadori product is oxidatively fragmented to give CML, among other products [181, 182]. In the “autoxidative glycosylation” pathway, CML is formed by the reaction between lysine and glyoxal, the latter of which results from metal-catalysed autoxidation of glucose by Wolff pathway [168, 183, 184]. In the third mechanism, glyoxal which can subsequently form CML is formed from fragmentation and oxidation of Schiff base via Namiki pathway [167, 185] (Scheme 6).

The nonenzymatic modification of amino groups in aminophospholipids also produces carboxymethyl compounds (Scheme 7), as CM-PE, whose generation in human erythrocytes and blood plasma has been demonstrated experimentally [145–147]. It is possible to hypothesize that the generation of CM-PE could proceed by similar mechanisms compared to CML, having as intermediate a dicarbonyl compound bound to PE, as it was proposed for N^*ε*^-(carboxymethyl)lysine formation by a hydroxyl radical mediate pathway (Scheme 6) [145, 148, 149]. Similar to its Amadori-PE precursor, AGE-PE is also hypothesized to trigger pathological processes [150, 151], being suggested useful as a potentially sensitive marker for reflecting hyperglycemic conditions in the early stage of diabetes [145, 152].

## 6. Conclusions

We reviewed some aspects related to nonenzymatic reactivity of membrane phospholipids, focusing on their roles as targets of reactions mediated by ROS or electrophilic compounds, such as RCS or reducing sugars, and their catalytic roles in some reactions that occurs on membrane surfaces. The formation of ALEs and AGEs has several intermediates and products characterized by their instability and differences in physicochemical properties. An example is glyoxal, a dicarbonyl intermediate which is generated by several pathways but rapidly generates other products, making their accurate quantification difficult* in vivo* [142]. In this context, theoretical methods which provide an atomistic-level description of these molecular systems are essential tools.

We described briefly some theoretical studies in order to gain insights about these processes at atomic level. These studies included calculus at DFT level of theory for obtaining mechanisms of reactions related to the formation of ALEs and AGEs using models of PE surface. Based on the results of these studies, it is possible to hypothesize that cell membrane phospholipids surface could enhance some reactions through a catalyst effect, and it could be a novel and accessory function of phospholipids surface into the cells. In their catalytic effect, the formation of hydrogen-bond networks between the phospholipids polar and charged groups and water molecules and a proton transfer is fundamental. These PE models were useful for obtaining insights about the reactivity on phospholipids surfaces, but if we desire to analyze the reactivity inside the membrane describing reactions as phospholipid peroxidation, the introduction of quantum mechanical/molecular mechanical (QM/MM) hybrid methods in these studies is desirable.

Other methods used for describing phospholipids bilayers were classical molecular dynamics simulations used in the study of formation of AGEs and ALEs for analysing the changes in the biophysical properties in the bilayer systems and the molecular interactions inside them generated by the introduction of some products of phospholipid peroxidation in the models or by their interaction with oxidant and reducing agents as ROS or sugars. In this point, the pathways of formation of ALEs and AGEs have common intermediates and products. The resulting ALEs and AGEs may be accumulated during aging and diabetes, introducing changes in cell membrane physicochemical and biological properties. Although the generation of RCS intermediates from lipids requires oxidative chemistry whereas nonenzymatic glycation may be either nonoxidative or oxidative, both pathways produce a number of carbonyl derivatives, which are responsible for the final production of ALEs and AGEs. Thus, although there are significant differences between the formation of ALEs and AGEs, many aspects of both pathways can be better understood if they are included in only one general carbonyl pathway that can be initiated by both lipids and carbohydrates [186]. More studies are desirable which could correlate the biophysics of modified phospholipids with metabolic studies in order to define the significance of the phospholipids properties for aging and the physiopathology of diseases such as diabetes, atherosclerosis, and Alzheimer.

## Figures and Tables

**Table 1:** Theoretical studies about reactivity on phospholipid surfaces at DFT level of calculus.

Studied reaction	Limited step	Calculated activation energy (Kcal mol^−1^)	Reference
Decomposition of hydrogen peroxide	Formation of an intermediate hydrogen trioxide	8.76	[20]

Schiff base formation between PE and acetaldehyde	Dehydration of carbinolamine intermediate	13.08	[19]

Amadori rearrangement from PE-D-erythrose Schiff base	Formation of a 1,2-enaminol intermediate	16.78	[22]

Carboxymethyl-PE formation from reaction between glyoxal and PE	Dehydration of Carbino-diol-amine intermediate	15.41	[23]

Reaction of aminoguanidine with dicarbonyl-PE product	Dehydration of an aminoguanidine adduct	—	[24]

**Table 2:** Some articles related to studies of oxidation of phospholipids which use molecular dynamics simulations.

Phospholipid molecular systems	Simulation time lipid force field	Aim	Reference
30 molecules of O_2_^−^, HO_2_, H_2_O_2_, HO, or O_2_ placed together at the aqueous phase surrounding a bilayer containing 128 2-oleoyl-1-palmitoyl-*sn*-glycero-3-phosphocholine (POPC) molecules.	50 ns Gromos 53A6	Studying the distribution, mobility, and permeation of ROS at phospholipid bilayers.	[25]

Coarse grained bilayer models containing different proportions of 1,2-dilinoleoyl-sn-glycero-3-phosphocholine (DUPC), cholesterol, and 1-palmitoyl-2-azelaoyl-sn-glycero-3-phosphocholine as oxidized lipid specie.	4–20 *μ*s Martini coarse grained	Evaluate a protector role of cholesterol on phospholipid bilayers from disruption caused by lipid oxidation.	[26]

Bilayers, each one containing cholesterol and 128 phospholipids with varying POPC to 1-palmitoyl-2-glutaryl-sn-glycero-3-phosphocholine (PGPC); the last is an oxidized lipid, with a truncated carbon chain terminated with a carboxylic acid.	100 ns Berger's nonpolarizable united-atom	Evaluate overall changes of the membrane structural and dynamical properties once they become oxidized.	[27]

2 molecules of 16-doxyl stearic acid randomly placed in each leaflet of a bilayer containing 62 phospholipid molecules each of 1-palmitoyl-2-(13-hydroperoxy-9,11-octadecanedienoyl)-lecithin and 1-palmitoyl-2-linoleoyl-glycero-3-phosphocholine (PLPC).	200 ns modified Berger	Studying lipid peroxidation effects on bilayers properties.	[28]

Bilayers containing 128 molecules of POPC and oxidized phospholipids PoxnoPC and PazePC in different proportions. PoxnoPC has a carbonyl group, and PazePC has an anionic carboxyl group pendant at the end of the short, oxidized acyl chain.	100 ns united-atom Berger	Evaluate the perturbation of overall membrane structural and dynamic properties by oxidatively modified phospholipids.	[29]

Bilayers containing 72 molecules of PLPC and oxidized PLPC (oxPLPC) molecules in different proportions. OxPLPC contained modified linoleoyl residues: 12-oxo-*cis*-9-dodecenoate and 13-hydroperoxy-*trans*-11, *cis*-9-octadecadienoate.	25 ns OPLS	Observing the effect of oxidized lipids on the sensitivity of a bilayer to electroporating fields.	[30]

PLPC bilayers containing 72 lipids, replacing 2, 4, 8, 18, and 36 PLPC lipid molecules with each oxidized lipid. The oxidized lipid was modeled by addition of a hydroperoxide group at position C9 or C13 of the linoleate tail and shifting the double bonds.	180 ns united-atom	Studying the effect of lipid peroxidation on the properties of PLPC bilayers.	[31]
